# Synergistic antitumor activity of the CD122-biased immunostimulatory cytokine NKTR-214 when combined with anti-PD-1 in murine tumor models

**DOI:** 10.1186/2051-1426-3-S2-P231

**Published:** 2015-11-04

**Authors:** Addepalli Murali, John L Langowski, Pena Rhoneil, Priyam Jha, Deborah H Charych, Stephen K Doberstein, Ute Hoch

**Affiliations:** 1 Nektar Therapeutics, Hyderabad, India; 2 Nektar Therapeutics, San Francisco, CA, USA

## Background

NKTR-214 is an immunotherapeutic drug that exerts its biological activity by binding and activating the interleukin-2 (IL-2) receptor beta subunit (IL-2Rb), thereby causing expansion of memory effector T cells in the tumor. NKTR-214 consists of 4-6 releasable polyethylene glycol (PEG) chains conjugated to IL-2 at a defined region within the protein. In vivo, some of these PEG chains slowly release to generate active IL-2 conjugates. The location of these PEG chains on IL-2 interferes with its interaction on the IL-2 receptor alpha (IL-2Ra), responsible for activating undesirable Treg cells in tumor. In vivo, the receptor bias markedly increases the ratio of tumor killing CD8 T cells to Treg cells in tumors, while simultaneously leading to high and sustained tumor exposure. NKTR-214 showed marked single agent efficacy in aggressive murine tumors, and synergy with anti-CTLA-4, producing durable responses. Here, we examine the efficacy and mechanism of NKTR-214 combined with anti-PD-1 in murine tumor models.

## Methods

Mice bearing subcutaneous CT26 colon, EMT6 mammary, or LLC Lewis Lung carcinoma tumors were treated with NKTR-214 i.v. every nine days (three administrations), murine anti-PD-1 twice-weekly for three weeks, or both in combination. Efficacy was determined based on tumor growth inhibition relative to vehicle (TGI), long-term tumor growth delay measured by time to reach 4x initial tumor volume (Tumor Volume Quadrupling Time, TVQT), and number of long term tumor-free animals.

## Results

In the CT26 model, single agent NKTR-214 led to greater TGI (66%) at day 11 than anti-PD-1 alone (37%). The combination proved synergistic with 9/10 animals tumor-free 100 days after dosing initiation. The EMT6 model proved more resistant to single-agent therapy, with NKTR-214 yielding 42% TGI at day 17. While TGI was not enhanced by addition of anti-PD-1, TVQT was increased to 29.6 days relative to single agent NKTR-214 (16.7) and vehicle (10.9), with 4/10 tumor free animals 73 days after dosing initiation. Finally, the LLC model proved sensitive to NKTR-214 single agent, with a 59% TGI at day 11 and 2/10 animals tumor free, while anti-PD-1 alone was not active. The combination of the two agents increased TVQT (20 days) compared to NKTR-214 alone (14), anti-PD1 alone (8) and vehicle (7), and increased tumor-free animals (4/10) relative to NKTR-214 (2/10) and anti-PD1 (1/10) alone.

## Conclusions

NKTR-214 provides an antibody-like dosing regimen and complementary mechanism of action to checkpoint inhibition. The synergy observed in mouse models holds promise for durable responses in humans.

